# Chondromyxoid Fibroma of the Proximal Fibula, a Rare Benign Bone Tumor With Unusual Location: A Case Report

**DOI:** 10.1155/cro/6015726

**Published:** 2025-11-23

**Authors:** Thanate Poosiripinyo, Sermsak Sukpanichyingyong, Krits Salang, Yium Thavornpitak, Thanapon Chobpenthai

**Affiliations:** ^1^Department of Orthopaedics, Khon Kaen Hospital, Khon Kaen, Thailand; ^2^Department of Pathology, Khon Kaen Hospital, Khon Kaen, Thailand; ^3^Princess Srisavangavadhana Faculty of Medicine, Chulabhorn Royal Academy, Bangkok, Thailand

## Abstract

**Case:**

An 11-year-old female presented with a painful mass in the right proximal leg. Radiographs revealed a geographic osteolytic lesion involving the metaphysis and extending into the diaphysis of the proximal fibula. She underwent extended curettage with adjuvant electrocautery and 95% ethanol for tumor ablation. The resulting defect was reconstructed using morselized talus allograft. At 18 months postoperatively, there was no evidence of local recurrence, bone remodeling was complete, and the patient achieved full functional recovery with an MSTS score of 100%, resuming unrestricted physical activity.

**Conclusion:**

This case highlights the diagnostic and therapeutic complexities associated with chondromyxoid fibroma (CMF) in atypical anatomical sites such as the proximal fibula. The use of extended curettage combined with adjuvant modalities and allograft reconstruction demonstrated favorable oncologic and functional outcomes. These findings contribute to the evolving evidence base for managing CMF in rare locations, underscoring the need for further studies to optimize treatment strategies.

## 1. Introduction

Chondromyxoid fibroma (CMF) is a rare, benign bone tumor, accounting for less than 1% of all bone tumors [1]. It is most commonly found in the proximal tibia and distal femur, with approximately 40% of cases occurring in these locations. The second most common site is the bones of the foot, accounting for 17% of cases [2]. However, the occurrence of this tumor in the fibula is extremely rare, with reports mostly limited to individual case studies [3–5]. Differentiating CMF from enchondroma and low-grade chondrosarcoma using radiographic and MRI imaging can be challenging, as these tumors can appear similar [6]. Accurate diagnosis requires histopathological examination, which is crucial since the treatment approaches for these conditions differ significantly.

The primary approach to managing CMF emphasizes complete excision with preservation of function. Due to its benign nature, wide resection is not routinely required unless the lesion is recurrent, aggressive, or located in anatomically complex regions where curettage may be insufficient [7]. For most cases, extended intralesional curettage remains the preferred approach, involving thorough mechanical removal of the tumor followed by adjuvant therapies such as phenol application, cryotherapy, or cementation to reduce recurrence risk [3, 8]. This method is less invasive and generally associated with lower morbidity compared to wide resection, which may be more applicable to deeply seated or structurally disruptive tumors. Recurrence rates after curettage vary, but with appropriate adjuvant use, outcomes are favorable and functional preservation is maximized. Another important consideration is how to fill the bone defect after the tumor has been removed. Options include autograft, allograft, bone substitutes, or bone cement [9]. Although allograft use in the treatment of CMF is not widely reported in the literature, it remains a viable option for managing large bone defects following tumor excision. Allografts offer several advantages, including abundant availability, effective defect filling, and promotion of osteoconduction and bone healing. These benefits are particularly relevant when autograft volume is insufficient or when harvesting autologous bone poses a risk of donor site morbidity [10]. However, allografts are associated with certain limitations, including higher cost, slower biological incorporation, and potential for minor immune responses or disease transmission, despite rigorous screening and sterilization protocols [11]. Several studies have reported an increased incidence of allograft failure or rupture, with a notably higher prevalence observed among younger patient populations [12, 13]. Allograft tissue is typically processed as either fresh-frozen (FF) or freeze-dried (FD), each with distinct biomechanical and immunological profiles. FF allografts retain superior structural integrity and biomechanical strength, especially in soft tissue applications, but may carry a slightly higher risk of immunogenicity due to preserved cellular components. In contrast, FD allografts are easier to store and handle, exhibit reduced immunogenicity due to cell devitalization, and may demonstrate faster incorporation in some cases, although their mechanical properties are generally inferior to FF grafts. Both types show comparable long-term fusion rates, and selection should be guided by defect size, anatomical location, and clinical context.

## 2. Case Report

An 11-year-old girl presented with a painful mass in the right proximal leg, particularly when bearing weight. She did not report any loss of appetite or weight loss. The mass was slow-growing. Physical examination revealed a poorly defined, fixed, and tender mass at the right proximal fibula, measuring 3 × 4 cm.

**Figure 1:**
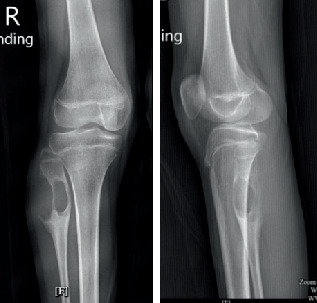
Biplanar plain radiographs of the right knee.

Plain radiographs revealed a geographic osteolytic lesion at the metaphysis extending to the diaphysis of the right proximal fibula, without invasion into the epiphysis or knee joint. The imaging demonstrated a laminated periosteal reaction on the posterior aspect of the tumor, with no evidence of matrix formation within the lesion (Figure 1).

**Figure 2:**
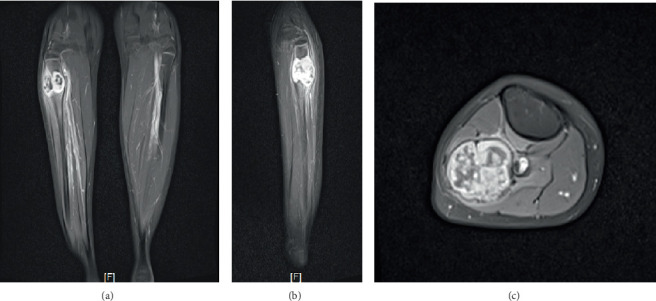
Magnetic resonance imaging. (a) Coronal view. (b) Sagittal view. (c) Axial view.

The MRI reveals a lobulated, well-defined, expansile intraosseous lesion involving the proximal metaphysis of the right fibula, with cortical expansion and thinning into the adjacent soft tissues, measuring approximately 2.9 × 2.9 × 4.0 cm (AP × width × length). The tumor exhibits a solid component with iso T1W signal, heterogeneous hyperintense T2W signal, internal punctate blooming foci, heterogeneous peripheral enhancement, and increased restricted diffusion (Figure 2).

**Figure 3:**
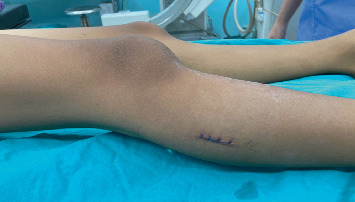
Incisional biopsy tract.

**Figure 4:**
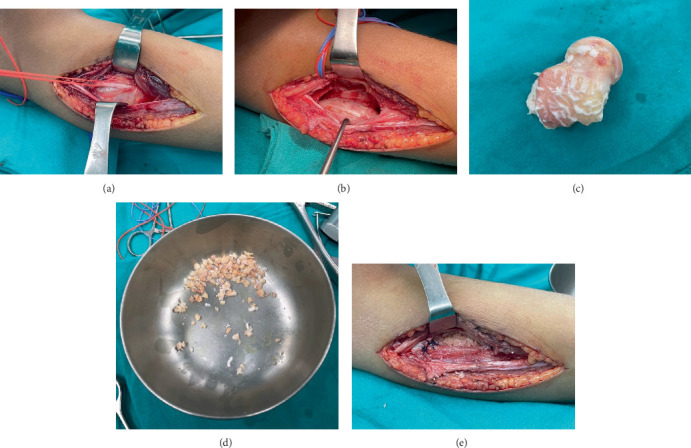
Operation. (a) Identify and protect the common peroneal nerve. (b) Bone defect after extended curettage. (c, d) Harvest of morselized allograft from the talus. (e) Filling the bone defect with morselized allograft harvested from the talus.

**Figure 5:**
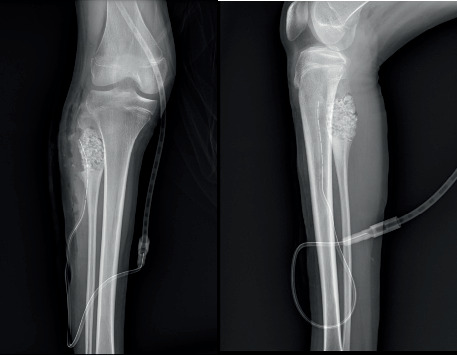
The postoperative plain radiographs demonstrate successful impaction of the allograft.

**Figure 6:**
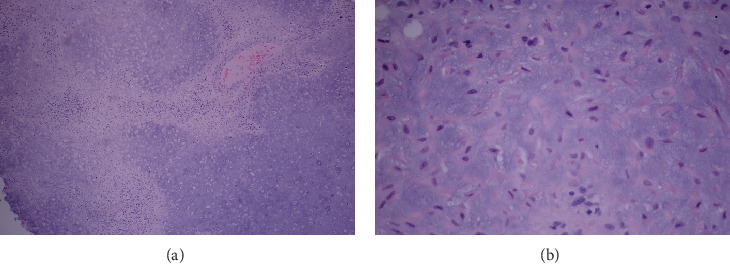
Histopathology report. (a) Low-power magnification shows multinodular appearance of chondromyxoid background (purple–blue hue). There are loose stellate cells scattered in the nodule. No tissue necrosis is seen. (b) High-power magnification shows stellate cells, displaying oval to spindle-shaped nuclei with bipolar or multipolar eosinophilic (red hue) extensions. Note the chondromyxoid background. No mitosis is seen.

An incisional biopsy was performed using a direct lateral approach over the lesion at the proximal fibula (Figure 3). The pathological report revealed bland stellate cells embedded within chondromyxoid material. The patient underwent extended curettage, followed by adjuvant treatment using electrocautery and 95% alcohol to ablate tumor cells. Following intralesional curettage, 10 cc of 95% ethanol was instilled into the cavity during each cycle as a chemical adjuvant, in accordance with previously published reports [14–16]. After curettage, the surgical cavity was soaked with 95% ethanol for 30–60 s, repeated across 3–4 cycles, and subsequently irrigated thoroughly with normal saline. Throughout the procedure, adjacent soft tissues were meticulously shielded using gauze or sterile drapes to prevent ethanol-related injury or contamination. Subsequently, the bone defect was filled using morselized allograft harvested from the talus (Figure 4). The postoperative plain radiographs demonstrate successful impaction of the allograft (Figure 5). The final pathological diagnosis is CMF (Figure 6).

**Figure 7:**
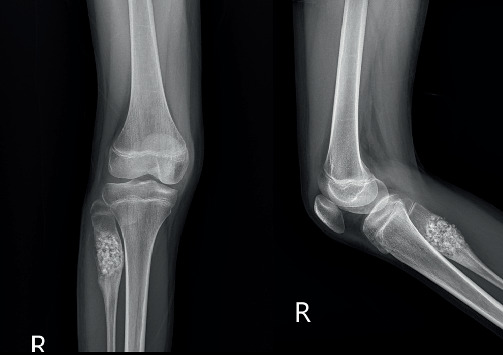
Plain radiographs at 2 months postoperation show successful incorporation of the allograft.

**Figure 8:**
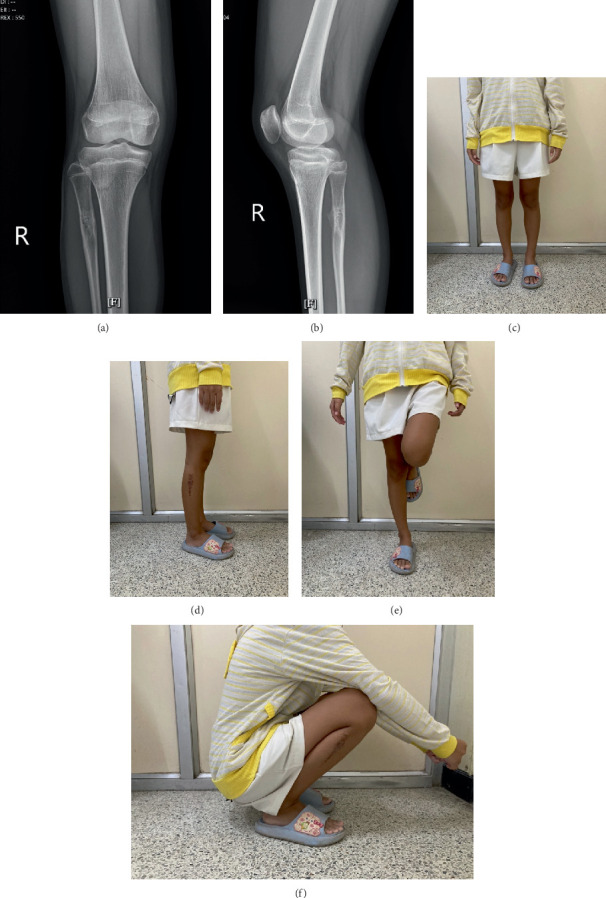
At 18 months postoperation. (a, b) Plain radiographs show excellent remodeling of the allograft. (c–f) Excellent functional outcomes with an MSTS score of 100%.

The patient received no additional adjuvant treatments. The postoperative course was uneventful, with no complications, and the patient was discharged within 1 week of surgery. At 2 months postoperation, the allograft had healed (Figure 7), and the patient achieved excellent functional outcomes with an MSTS score of 100%. The patient was able to run, jump, and engage in sports activities. At 18 months postoperation, there was no local recurrence, there was good bone remodeling at the proximal fibula, and the patient continued to demonstrate excellent functional outcomes with an MSTS score of 100% (Figure 8).

## 3. Discussion

CMF is a rare benign bone tumor, accounting for less than 1% of all bone tumors [1]. Its rarity and the unusual presentation in the proximal fibula, as in this case, make the diagnosis and management particularly challenging. CMF most commonly occurs in the proximal tibia and distal femur [17, 18], and involvement of the fibula is exceedingly rare [18, 19], with only isolated case reports in the literature [20, 21]. This unusual location can contribute to diagnostic difficulties, as clinical suspicion may be low when this tumor is present in atypical sites.

The occurrence of CMF in the fibula, particularly the proximal fibula, is rare and adds complexity to both diagnosis and treatment. As reported in this case, radiological findings such as osteolytic lesions with laminated periosteal reactions are typical of CMF but are not pathognomonic, making differentiation from other benign or low-grade malignant bone tumors, such as enchondroma or low-grade chondrosarcoma, essential. The rare location in the fibula further complicates radiographic interpretation and reinforces the importance of histopathological confirmation for an accurate diagnosis.

Given the benign nature of CMF, the primary treatment objective is complete tumor removal to prevent recurrence. Surgical options generally include extended curettage [3, 8] or wide resection [7]. Wide resection is associated with a lower recurrence rate but is technically more demanding and carries a higher risk of complications [22], particularly in cases where the tumor is located in weight-bearing bones or adjacent to critical neurovascular structures. Tomazini et al. reported the case of a 16-year-old male who presented with a CMF in the distal fibula. The patient initially underwent extended curettage; however, tumor recurrence was noted 1 year later. A wide resection of the distal fibula was subsequently performed, followed by reconstruction using an autogenous iliac bone graft. At the 14-month postoperative follow-up, no evidence of recurrence was observed [4]. In this case, extended curettage served as a less invasive approach, and the adjunctive use of electrocautery and 95% ethanol contributed to reducing recurrence while limiting morbidity. This method may be useful in managing tumors near the proximal fibula; however, when the fibular head and its ligamentous attachments are preserved and dissection remains within the periosteal plane, bone stability and neurovascular integrity are typically maintained.

After tumor removal, the decision to address the bone defect should be guided by its location, size, and functional relevance. In cases where the fibular head and periosteum are preserved, reconstruction may be considered in select cases to support early rehabilitation or address large defects. Options for defect filling include autografts, allografts, or synthetic bone substitutes [9]. Karaca et al. reviewed and reported on 31 cases of CMF. Of the 27 cases that underwent intralesional curettage, two involved filling the bone defect in the femur with allograft after curettage. Both cases achieved good outcomes without recurrence [9]. In this case, morselized allograft was chosen to fill the defect. Allografts present distinct advantages in pediatric patients, where autograft availability may be limited. Their ready availability allows for effective reconstruction of sizable defects, while avoiding donor site morbidity. In this case, complete healing and remodeling of the proximal fibula, along with a 100% MSTS score, underscore the efficacy of allograft use in managing postcurettage bone defects in CMF. Although the patient demonstrated satisfactory bone consolidation and functional recovery within 2 months postoperatively, CMF has a known potential for late recurrence, even years after surgical intervention [23]. Therefore, long-term radiological surveillance remains essential to detect any recurrence early and ensure sustained clinical outcomes beyond the initial follow-up period.

This case highlights the challenges in diagnosing and treating CMF, particularly when it arises in unusual locations like the proximal fibula. Extended curettage with adjuvant therapy, followed by allograft reconstruction, has emerged as a safe and effective treatment modality for CMF, particularly when the lesion is benign and located in anatomically sensitive regions such as the proximal fibula. Unlike en bloc resection, which may compromise surrounding structures and lead to functional deficits, curettage confined within the periosteal layer minimizes surgical morbidity while preserving critical soft tissue attachments and neurovascular integrity [7]. Adjuvant measures such as high-speed burring, phenol application, or cryotherapy enhance local tumor control by eliminating residual microscopic disease. The use of allograft to fill the resulting bone defect offers structural support and promotes osteointegration, especially in cases where autograft volume is insufficient or donor site morbidity is a concern [24]. Although allografts carry a theoretical risk of immune response or disease transmission, modern processing techniques including freeze-drying and irradiation have significantly reduced these risks while maintaining acceptable biomechanical properties [25]. In this case, the combination of extended curettage, adjuvant therapy, and allograft reconstruction yielded excellent functional outcomes and prevented recurrence, reinforcing the safety and efficacy of this approach. Given the rarity of CMF and the absence of standardized treatment protocols, especially for lesions in locations like the proximal fibula, this case contributes meaningful insight to the evolving literature and highlights the need for further research to optimize surgical strategies.

## Data Availability

The data that support the findings of this study are available on request from the corresponding author. The data are not publicly available due to privacy or ethical restrictions.
